# (±)-3-Deoxyradicinin Induces Stomata Opening and Chloroplast Oxidative Stress in Tomato (*Solanum lycopersicum* L.)

**DOI:** 10.3390/ijms24108467

**Published:** 2023-05-09

**Authors:** Simone Samperna, Clarissa Zanotti, Patrizia Scafato, Angela Boari, Sabina Visconti, Maurizio Vurro, Stefano Superchi, Antonio Evidente, Mauro Marra

**Affiliations:** 1 Department of Biology, University of Rome Tor Vergata, 00133 Rome, Italy; simone.samperna@libero.it (S.S.); zanotti.clarissa97@gmail.com (C.Z.); visconti@uniroma2.it (S.V.); 2 Department of Sciences, University of Basilicata, Via dell’Ateneo Lucano 10, 85100 Potenza, Italy; patrizia.scafato@unibas.it (P.S.); stefano.superchi@unibas.it (S.S.); 3 Institute of Sciences of Food Production, National Research Council, 70126 Bari, Italy; angela.boari@ispa.cnr.it (A.B.); maurizio.vurro@ispa.cnr.it (M.V.); 4 Department of Chemical Sciences, University of Naples Federico II, 80126 Naples, Italy; evidente@unina.it

**Keywords:** phytotoxins, radicinin, (±)-3-deoxyradicinin, bioherbicides, sustainable weed management, chloroplast oxidative stress, stomata opening, plant PCD

## Abstract

Radicinin is a phytotoxic dihydropyranopyran-4,5-dione isolated from the culture filtrates of *Cochliobolus australiensis,* a phytopathogenic fungus of the invasive weed buffelgrass (*Cenchrus ciliaris*). Radicinin proved to have interesting potential as a natural herbicide. Being interested in elucidating the mechanism of action and considering radicinin is produced in small quantities by *C. australiensis*, we opted to use (±)-3-deoxyradicinin, a synthetic analogue of radicinin that is available in larger quantities and shows radicinin-like phytotoxic activities. To obtain information about subcellular targets and mechanism(s) of action of the toxin, the study was carried out by using tomato (*Solanum lycopersicum* L.), which, apart from its economic relevance, has become a model plant species for physiological and molecular studies. Results of biochemical assays showed that (±)-3-deoxyradicinin administration to leaves induced chlorosis, ion leakage, hydrogen peroxide production, and membrane lipid peroxidation. Remarkably, the compound determined the uncontrolled opening of stomata, which, in turn, resulted in plant wilting. Confocal microscopy analysis of protoplasts treated with (±)-3-deoxyradicinin ascertained that the toxin targeted chloroplasts, eliciting an overproduction of reactive singlet oxygen species. This oxidative stress status was related by qRT-PCR experiments to the activation of transcription of genes of a chloroplast-specific pathway of programmed cell death.

## 1. Introduction

**Figure 1:**
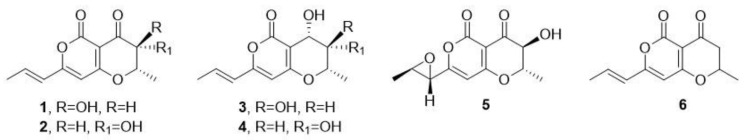
The structures of radicinin, 3-*epi*-radicinin, radicinol, 3-*epi*-radicinol, and cochliotoxin (**1**–**5**) isolated from *Cochliobolus australiensis* and (±)-3-deoxyradicinin (**6**) obtained through a novel and total synthesis.

Searching for novel natural compounds with herbicide potential against buffelgrass (*Pennisetum ciliare* L.), the investigation of the liquid culture of two of its foliar pathogens, *Cochliobolus australiensis* and *Pyricularia grisea*, led to the purification and identification of the known phytotoxin radicinin, which is from a new dihydropyran-pyran-4,5-dione named cochliotoxin, and of three structurally related metabolites, i.e., 3-epi-radicinin, radicinol, and 3-epi-radicinol (Figure 1).

Radicinin was originally isolated from the fungus *Stemphylium radicinum* [1] and has also been found to be produced by many other fungal species, such as *Cochliobolus lunatus* [2], *Phoma andina* [3], *Curvularia* sp. [4], and different species of the *Alternaria* genus [5,6,7]. Its chemical structure was first proposed in 1964 [8] and definitely determined in 1982 by X-ray crystallography [5] and recently by chiroptical spectroscopy [9]. From a biological point of view, radicinin is an interesting metabolite, having several biological effects, including promising herbicidal effects [5,10,11,12] and antifungal, insecticidal, and plant growth regulatory activities [13,14,15,16], as well as antibiotic activity against Gram-positive bacteria [17]. More recently, the toxin was shown to possess in vitro anticancer activity [18]. However, despite its interesting biological properties and its potential as a natural herbicide, the modest amounts obtainable by fungal fermentation constitute a bottleneck for scientific investigations or applicative developments. Additionally, for this reason, no research has been carried out to elucidate the mechanism of action of radicinin at the plant cellular level.

Recently, (±)-3-deoxyradicinin (DOR), the racemic form of (*S*)-3-deoxyradicinin, a biosynthetic precursor of radicinin [19], was obtained with a very good yield through a novel synthetic strategy (Figure 1) [20]. Synthetic DOR showed a phytotoxic activity very similar to that of the natural radicinin. In other studies, DOR, obtained by a different synthetic process, was shown to have biological effects similar to those available by radicinin, such as inhibition of growth of the bacteria *Xylella fastidiosa* and *Liberibacter crescens*, responsible for Pierce’s disease in grapevines and citrus huanglongbing, respectively [21,22]. Additionally, the anticancer activities of radicinin and DOR were found to be comparable [18]. Considering the possibility of having the synthetic DOR in larger amounts than the natural radicinin and the similar phytotoxic macroscopic effects of the two compounds, it was considered interesting to test the synthetic DOR in a number of successive and related bioassays and to perform molecular analyses to start elucidating its mechanism of action in plants and help evaluate its applicative potential on a rational basis. In this regard, the investigation was carried out on tomato (*Solanum lycopersicum* L.), which, apart from its economic relevance, is one of the best studied dicotyledonous plants, often used as a model system for plant research, for which genetic and molecular resources are available.

Results showed that the toxin caused, on tomato leaves, different phytotoxic effects, including uncontrolled stomata opening, leading to plant wilting, as well as ion leakage, hydrogen peroxide production, membrane lipid peroxidation, and chlorophyll loss, while on protoplasts, it determined ROS overproduction inside chloroplasts, triggering a chloroplast-mediated type of PCD.

## 2. Results

### 2.1. DOR Inhibited Root Growth and Induced Spot Lesions, Leaf Chlorosis, and Chlorophyll Loss in Tomato Leaves

**Figure 2:**
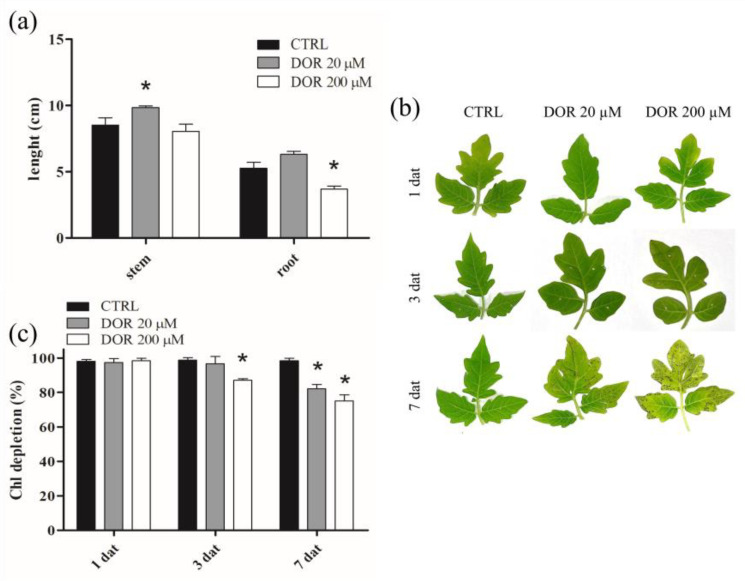
Stem and root lengths (**a**), leaf spot lesions and chlorosis (**b**), and chlorophyll content of tomato seedlings or leaves treated with DOR (**c**). (**a**) Plants were grown in vitro in MS medium supplemented, or not, with 20 or 200 μM DOR. Stem and root lengths were measured 7 days after treatment (dat). (**b**) Leaves of two-week-old tomato plants were detached and inoculated with 5 μL droplets of a solution containing 20 or 200 μM DOR, and the formation of spot lesions and chlorotic areas were observed starting 1 day after treatment. (**c**) Leaves of two-week-old tomato plants, sprayed with a solution containing 20 or 200 μM DOR, were collected after 1, 3, and 7 days after treatment, and chlorophyll content was evaluated, as reported in Section 4.4. Results from three independent experiments are reported; values are expressed as the mean ± SEM. Statistical significance was attributed by Student’s test (* *p* < 0.05).

The effect of DOR on the growth of tomato plants was investigated by treating ten-day-old *Solanum lycopersicum* seedlings with 20 or 200 μM concentrations of the toxin. DOR supplemented at 20 μM had a slight growth-stimulating effect on both stems and roots (Figure 2a). On the contrary, at 200 μM, the toxin significantly hampered root growth. The phytotoxicity of DOR was also tested on leaves. The application of 5 μL droplets containing 20 μM or 200 μM DOR on two-week-old detached leaves induced spot lesions 3 days after application. These symptoms increased depending on both toxin concentration and time of application; extensive chlorosis, along with several brown-spot lesions, were evident in leaves treated with 200 μM DOR after 7 days (Figure 2b). These results were confirmed by chlorophyll content analysis. DOR-treated leaves showed a reduction in their chlorophyll content, with a concentration and time dependence similar to that of foliar lesions (Figure 2c).

### 2.2. DOR Induced Stomata Opening and Wilting of Tomato Plants

**Figure 3:**
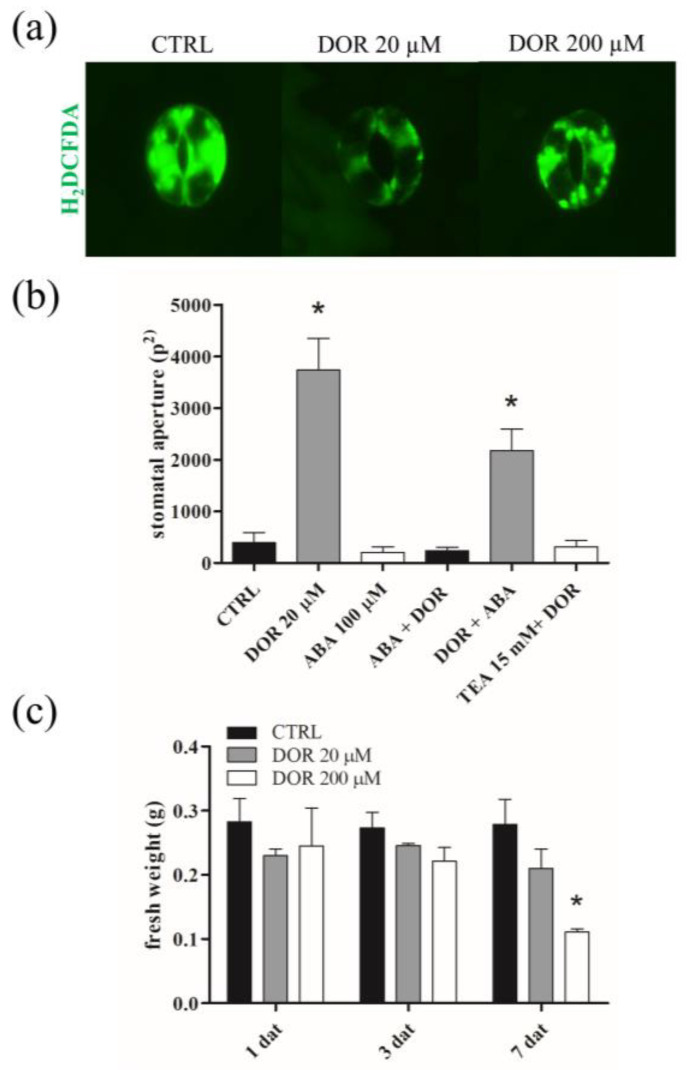
Fluorescence microscopy images of stomata treated with DOR (**a**), degree of stomata opening after treatments with DOR, ABA, and TEA (**b**), and fresh weight of tomato seedlings treated with DOR (**c**). (**a**) Stomata opening assay was performed on leaves detached from two-week-old tomato plants and incubated in the dark with 20 μM or 200 μM DOR for 1 day. After treatment, the leaf lower epidermis was detached by sticking it to adhesive tape; it was incubated with 10 μM H_2_DCFDA for 5 min and observed under a fluorescence microscope. (**b**) Leaves detached from two-week-old tomato plants were incubated in the dark for 1 day with the compounds and at concentrations indicated on the x-axis. Where two compounds are indicated, the first one was administered before, for 1 day, in the dark, at the same concentrations used for single administration. The stomata opening assay was performed as in (**a**). Leaf epidermis was observed under an optical microscope. The degree of stomata opening was estimated as pixels^2^, using the Image J software. (**c**) Two-week-old tomato seedlings were cut 2 cm over the roots and immersed in a solution containing, or not containing, 20 μM or 200 μM DOR. At indicated times of treatment, seedlings were collected, and fresh weight was measured. Results from three independent experiments are reported; values are expressed as the mean ± SEM. Statistical significance was attributed by Student’s test (* *p* < 0.05).

Plant–pathogen interactions may involve the induction of stomata opening, which is exploited by the pathogen to penetrate the leaf mesophyll with infective hyphae or to determine uncontrolled transpiration leading to leaf wilting [23,24]. Therefore, we tested, using the epidermal peel assay, whether DOR administration on tomato leaves was able to cause the opening of the stomata. The effect was visible after 6 h and was maximal 1 day after treatment at a concentration of 20 μM DOR (Figure 3b). DOR was ineffective when stomata were pre-treated with the stomata closure-inducing hormone abscisic acid (ABA) [25] or with the K^+^ channel blocker tetraethylammonium chloride (TEA), while ABA could partially overcome the effect of pre-treatment with DOR (Figure 3b). Since it is known that stomata closure induced by different stimuli, including ABA, is mediated by ROS generation inside guard cells, we incubated the stomata with the ROS probe 2′,7′-dichlorodihydrofluorescein diacetate (H_2_DCDFA) after the treatment with the toxin. Stomata opening induced by the toxin was accompanied by a significant reduction in ROS generation inside guard cells, compared to the control sample (Figure 3a). It is noteworthy that 200 μM DOR, which was less effective than 20 μM DOR in inducing stomata opening, was also less effective in reducing ROS levels. These results prompted us to perform a seedling wilting assay to verify whether DOR-induced stomata opening could result in the increased transpiration and wilting of tomato plants in vivo. DOR induced a significant loss of fresh weight, particularly at 200 μM, 7 days after administration (Figure 3c).

### 2.3. DOR Induced Ion Leakage, Hydrogen Peroxide Production, Membrane Lipid Peroxidation, and Callose Deposition in Tomato Leaves

**Figure 4:**
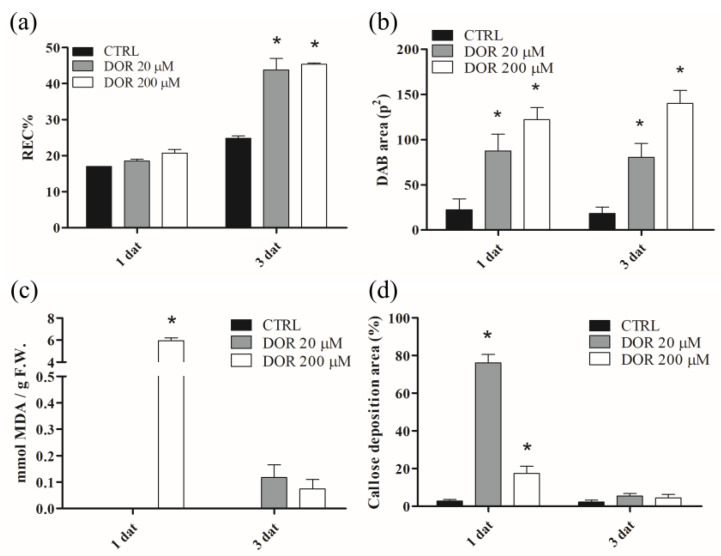
Ion leakage (**a**), H_2_O_2_ production (**b**), MDA production (**c**), and callose deposition (**d**) in tomato leaves treated with 20 or 200 μM DOR. Leaves of two-week-old tomato plants were exhaustively sprayed with a solution containing 20 or 200 μM DOR and collected 1 and 3 days after treatment (dat). REC%, DAB production, MDA content, and callose deposition were estimated, as reported in Section 4.5, Section 4.6, Section 4.7 and Section 4.8, respectively. Results from three independent experiments are reported; values are expressed as the mean ± SEM. Statistical significance was attributed by Student’s test (* *p* < 0.05).

To assess whether DOR could bring about cellular damages, as well as to elicit defense responses, biochemical assays were performed on tomato leaves treated with 20 or 200 μM DOR to evaluate membrane ion leakage, hydrogen peroxide production, membrane lipid peroxidation, as well as callose deposition. The relative electric conductivity (REC%), which is an indirect estimate of membrane damage, was increased by 200 μM DOR 1 day after treatment and by 20 μM DOR 3 days after treatment (Figure 4a). H_2_O_2_ is the most stable ROS species induced by pathogens in plants; it can be detected in situ by the 3,3′diaminobenzidine (DAB) chromogen assay. DOR induced hydrogen peroxide production 1 day after treatment, and the production was maintained after 3 days (Figure 4b). The effect was concentration-dependent from 20 to 200 μM DOR. The corresponding images of leaves treated with DOR and stained with DAB are reported (Figure S1a). Malondialdehyde (MDA) is produced from membrane lipid oxidation by ROS, thereby representing an indirect way of estimating oxidative membrane damage. At 20 μM, DOR was almost ineffective, whereas at 200 μM, the toxin significantly induced lipid peroxidation. The effect was strongly reduced after 3 days (Figure 4c).

Callose deposition in the cell wall is a component of the basal defense common to many plants, aimed at limiting pathogen progression. Other than fungal infection, it can also be induced by phytotoxins. The 20 μM DOR strongly induced callose deposition 1 day after treatment. The effect was less evident upon treatment with DOR 200 μM and was lost after 3 days at both DOR concentrations (Figure 4d). The corresponding images of leaves treated with DOR and stained with aniline blue for callose detection are shown (Figure S1b).

### 2.4. DOR Reduced Cell Viability and Induced Oxidative Stress in Chloroplasts from Tomato Leaf Protoplasts

**Figure 5:**
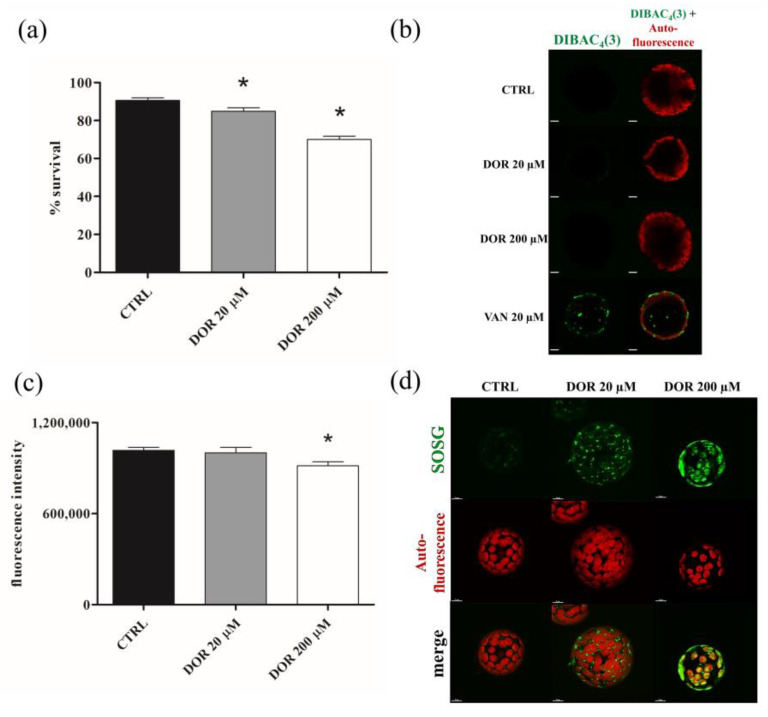
Cell viability (**a**), confocal microscopy imaging of plasma membrane potential (**b**), chlorophyll autofluorescence intensity (**c**), and confocal microscopy imaging of singlet oxygen generation inside chloroplasts (**d**) of DOR-treated protoplasts from tomato leaves. (**a**) Protoplasts from leaves of two-week-old tomato plants were treated with 20 or 200 μM DOR for 1 h, incubated with 3 μM FDA (**a**), 1 μM DIBAC_4_ (3) (**b**), and 5 μM SOSG (**d**), and observed under a fluorescence microscope (**a**) or under a confocal microscope (**b**,**d**). Results from three independent experiments are reported; values are expressed as the mean ± SEM. Statistical significance was attributed by Student’s test (* *p* < 0.05).

The effect of DOR on cell viability was evaluated using fluorescein diacetate (FDA) staining of protoplasts prepared from tomato leaves. Incubation with 20 and 200 μM DOR for 60 min reduced protoplast viability, and the effect was dose-dependent (Figure 5a). In order to identify possible sites of DOR action at the subcellular level, confocal microscopy experiments were performed on protoplasts incubated with micromolar concentrations of the toxin. The morphology and/or functionality of cellular organelles were monitored by means of specific fluorescent dyes.

Plasma membrane potential was monitored using *Bis*-(1,3-dibutylbarbituric acid) trimethine oxonol (DIBAC_4_ (3)), which accumulates inside the cell, depending on its transmembrane potential. The probe did not accumulate in the untreated, 20, or 200 μM DOR-treated protoplasts, whereas it accumulated in vanadate-treated, depolarized protoplasts (Figure 5b). Since DIBAC_4_ (3) enters depolarized cells, exhibiting enhanced fluorescence, our results indicated that the toxin did not induce plasma membrane potential depolarization. Mitochondrial network integrity and functionality were investigated by using MitoTracker Red, which accumulates into functional mitochondria, depending on their membrane potential, and MitoSOX Red, which becomes highly fluorescent upon oxidation by mitochondrial oxygen superoxides. The results were negative. No alterations of the mitochondrial network or ROS over-production were observed after incubation with 20 or 200 μM DOR (Figure S2a,b, respectively). Chloroplast functionality was investigated by evaluating chlorophyll autofluorescence and using the probe Singlet Oxygen Sensor Green (SOSG). DOR reduced chloroplast autofluorescence in a dose-dependent manner (Figure 5c), suggesting a possible detrimental effect of the toxin on organelle functionality. This indication was confirmed by using the SOSG probe. SOSG is highly selective for singlet oxygen (^1^O_2_), and chloroplasts are the main source, in the plant cell, of singlet oxygen, which is produced during photosynthesis and over-produced when the functionality of the organelle is hampered. Incubation with 20 and 200 μM DOR induced singlet oxygen over-production (Figure 5d). The effect was proportional to the toxin concentrations and indicated that DOR hampered the functionality of chloroplasts. Vacuole status was monitored using acridine orange (AO), which accumulates in acidic compartments. No differences between control and DOR-treated protoplasts were observed. In both samples, a diffuse green fluorescence, corresponding to the cellular volume occupied by intact vacuoles, was detected (Figure S2c), thereby demonstrating that the toxin did not alter the integrity of the organelle.

### 2.5. DOR Determined DNA Fragmentation in Protoplasts and Elicited the Transcription of Chloroplast-Induced Programmed Cell Death (PCD) Genes in Tomato Leaves

**Figure 6:**
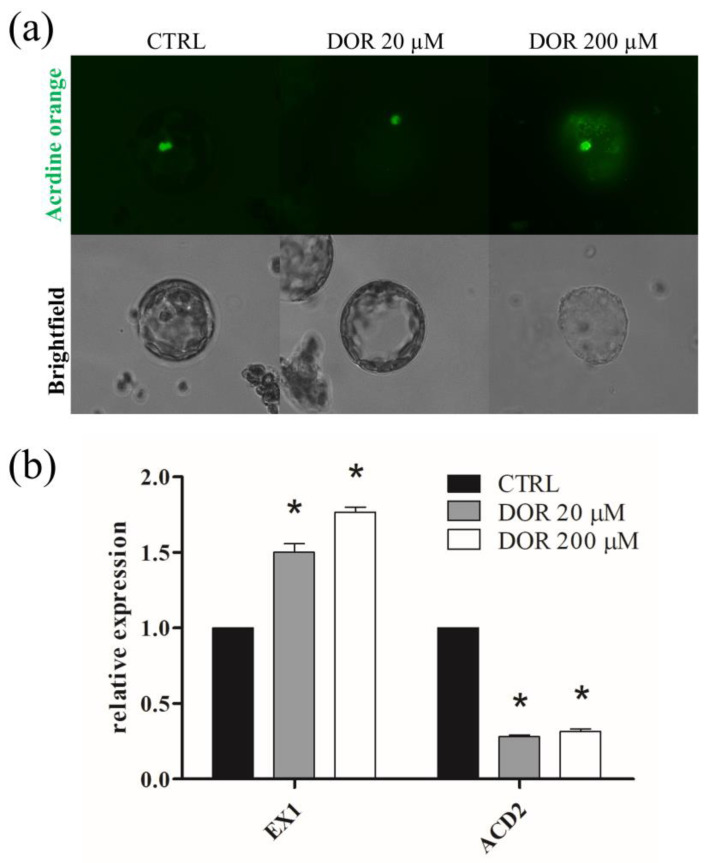
Fluorescence microscopy of protoplasts treated with DOR and stained with AO (**a**) and qRT-PCR analysis of chloroplast-induced PCD regulatory genes (**b**). (**a**) Protoplasts from tomato leaves were treated with 20 or 200 μM DOR for 1 h, then incubated with 3 μM AO for 5 min and observed under a fluorescence microscope). (**b**) Leaves of two-week-old tomato plants were sprayed with a solution containing 20 or 200 μM DOR and collected 3 days after treatment. Total mRNA was extracted, and the qRT-PCR analysis of relative expressions of EX1 and ACD2 genes were performed, as described in Section 4.9. Results from three independent experiments are reported; values are expressed as the mean ± SEM. Statistical significance was attributed by Student’s test (* *p* < 0.05).

In order to ascertain whether DOR treatment resulted in PCD induction, we stained protoplasts with AO to detect nuclear DNA fragmentation, which is one of the markers of cell death. AO binds to DNA, emitting a green fluorescence that becomes more intense and green-yellow upon chromatin fragmentation [26]. DOR, particularly at 200 μM, determined a more intense and yellow-shifted AO fluorescence in the nuclei of protoplasts (Figure 6b), thus indicating that toxin administration elicited PCD. Singlet oxygen overproduction inside the chloroplast can act as a signal activating a specific pathway of PCD mediated by the EXECUTER1 (EX1) gene [27]. Given the observed effect of DOR on chloroplast ^1^O_2_ overproduction, the induction of EX1 gene transcription after DOR administration on tomato leaves was analyzed by qRT-PCR, together with that of the ACCELERATED CELL DEATH 2 (ACD2) gene, which is considered a pro-survival regulator of the same pathway of PCD [28,29].

The EX1 gene was up-regulated in a dose-dependent manner by 20 and 200 μM DOR after 3 days; conversely, DOR treatment resulted in the down-regulation of the ACD2 gene (Figure 6a). These results, taken together, suggest that DOR was able to bring about oxidative stress in chloroplasts, which, in turn, induced PCD through the modulation of the transcription of genes of a chloroplast-specific pathway.

## 3. Discussion

Phytotoxins, which have been selected to interfere with pivotal processes of plant cells, possess many peculiar biological properties, representing novel compounds that can be exploited as antibiotics or bioherbicides or as drugs for the treatment of human diseases. However, despite their potential, with few exceptions, very little is known about their molecular effects in plant (and animal) cells, information that is desirable for developing more focused applications. In this study we investigated the mechanism of action in tomato plants of DOR, a synthetic analogue of the phytotoxin radicinin, which showed similar phytotoxicity and is available in suitable amounts. Two-hundred μM DOR reduced the growth of both stems and roots of tomato seedlings. This finding is in line with the similar growth-inhibitory effects of radicinin on carrots [16] and *Amaranthus retroflexus* roots [17] at comparable concentrations and confirms that these compounds act as nonspecific phytotoxins.

Micromolar concentrations of DOR were toxic to tomato leaves, producing chlorosis associated with chlorophyll loss and brown-spot lesions in dose- and time-dependent manners. The toxicity in leaves was related to the induction of hydrogen peroxide production, ion leakage, and lipid peroxidation, thus suggesting that the toxin was able to alter cell ROS homeostasis, resulting in altered membrane functionality. These biochemical events can provide a rationale for the mechanism of general phytotoxicity in plant tissues.

DOR induced stomata opening. The effect was inhibited by specific modulators of stomata, such as TEA or ABA, and, intriguingly, was accompanied by a decrease in guard cells of the ROS, which function as signaling intermediates in the mechanism of stomata closure induced by different stimuli, including ABA. The uncontrolled opening of the stomata caused the wilting of tomato plants. This finding provides evidence of a further mechanism of phytotoxicity acting specifically on guard cells, involving the modulation of ROS as signaling intermediates of stomata movements and resulting in toxicity at the whole plant level by wilting. The effect is reminiscent of that of fusicoccin, a deeply investigated, wilt-inducing phytotoxin, whose mechanism of stomata opening induction involves the activation of the plasma membrane H^+^-ATPase mediated by 14-3-3 proteins [23,24] and deserves further molecular investigation.

Confocal microscopy experiments on protoplasts demonstrated that DOR specifically targeted chloroplasts, where it caused singlet oxygen overproduction, whereas it did not hamper plasma membrane potential or alter mitochondrial functionality and vacuole integrity.

In plant and animal cells, ROS overproduction determines the oxidation of cellular components and the induction of PCD. In plants, the oxidative burst and PCD are also part of the basal defense response, which necrotrophic pathogens exploit to kill cells and derive nutrients [30]. In this respect, fungal phytotoxins have been shown to be able, per sè, to trigger the defense response and to induce PCD [31,32]. DOR induced DNA fragmentation in the nuclei of protoplasts, a characteristic feature of PCD. This finding strongly suggests that the toxin was able to elicit PCD in the plant cell by determining an oxidative stress status within the chloroplasts involving singlet oxygen overproduction. To corroborate this hypothesis, the involvement of a recently discovered chloroplast-mediated pathway of PCD triggered by the perturbation of ^1^O_2_ homeostasis and involving the activation of specific genes [33] was investigated using qRT-PCR. Results indicate that DOR in tomato leaves up-regulated the transcription of the pro-death EX1 gene and down-regulated that of the pro-survival ACD2 gene of the chloroplast-specific pathway of PCD. These pieces of evidence support a model that involves the specific targeting of DOR to chloroplasts, where the toxin hampers membrane functionality, causing singlet oxygen overproduction, which, in turn, determines the PCD of cells and tissue toxicity.

## 4. Materials and Methods

### 4.1. (±)-3-Deoxyradicinin Preparation

The (±)-3-deoxyradicinin used in the present study was obtained from total synthesis, as previously described [20]. Purity (>98%) was ascertained by HPLC and ^1^H NMR analysis.

### 4.2. Plant Growth and Treatments

Cherry tomato (*Solanum lycopersicum* L.) seeds (Blumen Vegetable Seeds, Piacenza, Italy) were layered on cheesecloth soaked with a nutrient solution, prepared according to [34], and cultivated with the same solution in hydroponics and in a climatic chamber (VB1514 Vötsch, Rosenfeld, Germany) at 22 °C and 80% humidity, with a 16/8 h light/dark cycle, for two weeks. For DOR treatments, two-week-old plants were sprayed with 20 or 200 μM solutions of (±)-3-deoxyradicinin containing 0.05% Tween 20 until a complete wetting was observed. Where specified, administration of DOR was performed by dipping fully expanded young leaves from two-week-old plants in solutions containing DOR. For stomatal aperture analysis, leaves detached from two-week-old tomato plants were incubated in the dark, with 20 or 200 μM DOR for 1 day. Pre-treatments with 100 μM ABA or with 15 mM TEA were carried out in the dark for 1 day. After treatments, leaves were removed from solutions and carefully dried, and the lower epidermis was detached by sticking it to adhesive tape, as previously described [35]. After incubation in a solution containing 10 μM H_2_DCDFA for 5 min in the dark, it was observed under a fluorescence microscope (ECLIPSE TE2000-E, Nikon, Melville, NY, USA)

### 4.3. Preparation of Protoplasts from Tomato Leaves

Protoplast purification was performed, as previously reported [36]. One hundred mg of leaves from two-week-old tomato plants were cut into 0.5–1 mm strips, submerged into the enzyme solution (1% cellulase R-10, 0.25% macerozyme R-10, 0.4 M mannitol, 20 mM KCl, 20 mM MES-OH, 10 mM CaCl_2_, pH 5.7), incubated for 30 min under vacuum, and then incubated again for 150 min in the dark at 25 °C. After digestion, the mixture was filtered with a 100 μm cell strainer (Falcon REF352340, Corning, Deeside, UK) and centrifuged at 100× *g* for 2 min. The protoplast pellet was washed twice with 5 mL of ice-cold 2 mM MES-KOH buffer, pH 5.7, containing 154 mM NaCl, 125 mM CaCl_2_, and 5 mM KCl, and resuspended in 3 mL of 2 mM MES-KOH buffer, pH 5.7, containing 0.4 M mannitol and 15 mM MgCl_2_. The number of purified protoplasts was determined with a Thoma cell counting chamber. Purified protoplasts were utilized for confocal microscopy analysis, as reported in Section 4.10.

### 4.4. Total Chlorophyll Assay

The chlorophyll content of two-week-old tomato leaves, untreated or treated with 20 μM or 200 μM for 1 day or 3 days, was estimated, as described previously [36]. One hundred mg of control or DOR-treated leaves were collected in a Falcon tube with 5 mL of DMSO. After incubation at 65 °C for 90 min and subsequent cooling at 25 °C, samples were centrifuged at 3000× *g* for 5 min, the supernatant was recovered, and the chlorophyll content was estimated by measuring the absorption at 663 nm and 645 nm.

### 4.5. Ion Leakage Assay

Ion leakage assay was performed, as described previously [36]. Two hundred mg of leaves from two-week-old tomato plants, untreated or treated with 20 µM or 200 µM DOR for 1 day or 3 days, were cut into 5 mm strips and submerged in 30 mL of deionized water for 2 h at 25 °C. After incubation, the electrical conductivity was measured using a conductimeter. Measurements were reported as relative electrical conductivity (REC%). Boiled samples were used to determine the maximum percentage of electrolyte leakage, which was calculated using the following formula: REC% = C1/C2 *×* 100 (C1= conductivity at 25 °C; C2 = conductivity at 100 °C).

### 4.6. H_2_O_2_ Production Assay

H_2_O_2_ was detected in leaves from two-week-old tomato plants, untreated or treated with 20 µM or 200 µM DOR for 1 day or 3 days, by staining with DAB, as described previously [36]. For each sample, five leaves were submerged in a solution of 10 mM DAB, pH 6.8, containing 0.05% (*w*/*v*) Tween 20. After vacuum infiltration for 15 min and incubation for 5 min under stirring, leaves were submerged in a bleaching solution of ethanol:acetic acid:glycerol 3:1:1 (*v*/*v*/*v*) and boiled for 15 min to remove chlorophyll. After cooling at 25 °C, the bleaching solution was eliminated, fresh bleaching solution was added, and leaves were mounted on glass slides for optical microscopy observation. Quantitative analysis of pixels from leaf images was performed using ImageJ 1.51j8 software (LOCI, University of Wisconsin, Madison, WI, USA).

### 4.7. Membrane Lipid Peroxidation Assay

Membrane lipid peroxidation was estimated in leaves from two-week-old tomato plants, untreated or treated with 20 µM or 200 µM DOR for 1 day or 3 days, using the MDA method, as described previously [36]. One hundred mg of control or DOR-treated tomato leaves were homogenized in liquid N_2_, suspended in 500 μL of 0.1% trichloroacetic acid (TCA), and centrifuged at 15,000× *g* for 10 min at 4 °C. One hundred µL of the supernatant were added to 1.5 mL of 0.5% thiobarbituric acid in 20% TCA and incubated for 25 min at 95 °C. After incubation, the reaction was blocked by placing the samples in ice. After cooling at 25 °C, sample absorbance was measured at 532 nm and 600 nm.

### 4.8. Callose Deposition Assay

Leaves from two-week-old tomato plants, untreated or treated with 20 μM or 200 μM DOR for 1 day or 3 days, were collected and placed in Eppendorf tubes. Chlorophyll was removed by incubation in a solution of acetic acid:ethanol (1:3 *v*/*v*) overnight at 25 °C. After incubation, the solution was removed and replaced with a solution of 150 mM K_2_HPO_4_, pH 6.8, for 30 min. Leaves were then submerged in a solution of 0.01% (*w*/*v*) aniline blue in 150 mM K_2_HPO_4_, pH 6.8, for 2 h. After incubation, leaves were placed in 50% (*v*/*v*) glycerol and mounted on glass slides for observation with an optical/epifluorescent microscope (ECLIPSE TE2000-E, Nikon, Melville, NY, USA).

### 4.9. qRT-PCR Analysis of Genes Expression

For qRT-PCR analysis of gene expression, leaves from two-week-old tomato plants, untreated or treated with 20 µM or 200 µM DOR, were harvested 3 days after toxin administration. Total RNA was extracted from 100 mg of homogenized leaves using RiboZOL (vWR, Radnor, PA, USA). For cDNA synthesis, 20 μg of total RNA was retro-transcribed by using the FastGene Scriptase II cDNA kit (Nippon Genetics Europe, Düren, Germany), according to the manufacturer’s instructions, and stored at −80 °C until use. qRT-PCR experiments were performed, as previously described [35], using the LightCycler apparatus (Roche, Basel, Switzerland) and the SYBR GREEN dye (PCR Biosystems, London, UK). The 2^−ΔΔCt^ method was applied to evaluate the level of gene expression, using the ACT3 and UBI3 genes of *S. lycopersicum* as housekeeping genes. Results represent mean values ± SD of independent experiments (n = 3). Samples were run in technical triplicates. Statistical significance was attributed by Student’s test (*p* < 0.05). The primers used for amplification are listed in Supplementary Table S1.

### 4.10. Fluorescence and Confocal Microscopy

Confocal microscopy experiments were performed according to [36] by using protoplasts prepared from tomato leaves, as reported in Section 4.3. In all experiments, protoplasts were incubated with 20 µM or 200 µM DOR for 1 h before fluorescent dye staining and confocal microscopy observation. For cell viability determination, after treatment with DOR, protoplasts were incubated with 3 μM FDA for 5 min and observed under a fluorescence microscope (ECLIPSE TE2000-E, Nikon, Melville, NY, USA). For mitochondria imaging, 1 µM MitoTracker™ Red CMXRos (Thermo-Fisher Scientific, Waltham, MA, USA) at 579 excitation and 599 emission wavelengths, respectively, was used. To monitor mitochondrial ROS production, 3 µM MitoSOX^TM^ Red (Thermo-Fisher Scientific, Cambridge, UK) at 510 nm excitation and 580 nm emission wavelengths, respectively, was used. For vacuole visualization, 3 µM acridine orange (Sigma-Aldrich, St. Louis, MO, USA) at 488 nm excitation and 526 nm emission wavelengths, respectively, was used. To monitor singlet oxygen levels, 5 µM SOSG (Singlet Oxygen Sensor Green, Invitrogen, Waltham, MA, USA) at 504 excitation and 525 emission wavelengths, respectively, was used. To monitor plasma membrane potential, 1 µM DIBAC_4_(3) (Thermo-Fisher Scientific, Waltham, MA, USA) at 493 excitation and 516 emission wavelengths, respectively, was used. Images were acquired with a laser-scanning confocal microscope FV1000, Olympus (Hamburg, Germany), using a 60× oil objective (N.A.: 1.35) in z stack mode (step size: 0.45 μM). Images were processed using the Imaris 6.2.1 software (Bitplane, Zurich, Switzerland).

### 4.11. Statistical Analyses

Experiments were repeated three times. Data were expressed as the mean ± standard error of the mean (SEM). GraphPad Prism software 7 (GraphPad Software, Inc., San Diego, CA, USA) was used to test the significance of the data by unpaired *t*-Student’s test; *p* < 0.05 was used to indicate a statistically significant difference.

## 5. Conclusions

In this study, an investigation of the cellular and molecular determinants of (±)-3-deoxyradicinin phytotoxicity in tomato was carried out. Results of biochemical assays showed that in leaves, (±)-3-deoxyradicinin induced chlorosis, ion leakage, and hydrogen peroxide production, as well as membrane lipid peroxidation. In guard cells, the compound determined the uncontrolled opening of stomata, which, in turn, resulted in plant wilting. Analysis of protoplasts demonstrated that the toxin targeted chloroplasts, eliciting an overproduction of reactive singlet oxygen species and inducing DNA fragmentation, events that were related by qRT-PCR analysis to the modulation of the transcription of genes of a chloroplast-specific pathway of PCD.

## Data Availability

Not applicable.

## Supplementary Materials

The following supporting information can be downloaded at: https://www.mdpi.com/article/10.3390/ijms24108467/s1.

## Abbreviations

ABA: abscisic acid; ACD2: ACCELERATED CELL DEATH 2; AO: acridine orange; DAB: 3,3′diaminobenzidine; DIBAC_4_ (3): *Bis*-(1,3-dibutylbarbituric acid trimethine oxonol; DOR: (±)-3-deoxyradicinin; EX1: EXECUTER1; FDA: fluorescein diacetate; H_2_DCDFA: 2′,7′-dichlorodihydrofluorescein diacetate; MDA: malondialdehyde; PCD: programmed cell death; ROS: reactive oxygen species; SOSG: singlet oxygen sensor green; TEA: tetraethylammonium chloride.
