# Supplementary material for: (±)-3-Deoxyradicinin Induces Stomata Opening and Chloroplast Oxidative Stress in Tomato (Solanum lycopersicum L.)
Source: Int J Mol Sci. 2023 May 9;24(10):8467. doi: 10.3390/ijms24108467 (PMC10218477; doi:10.3390/ijms24108467)
Supplement: Supplementary file 1 [file ijms-24-08467-s001.zip › ijms-2357810-supplementary.pdf]

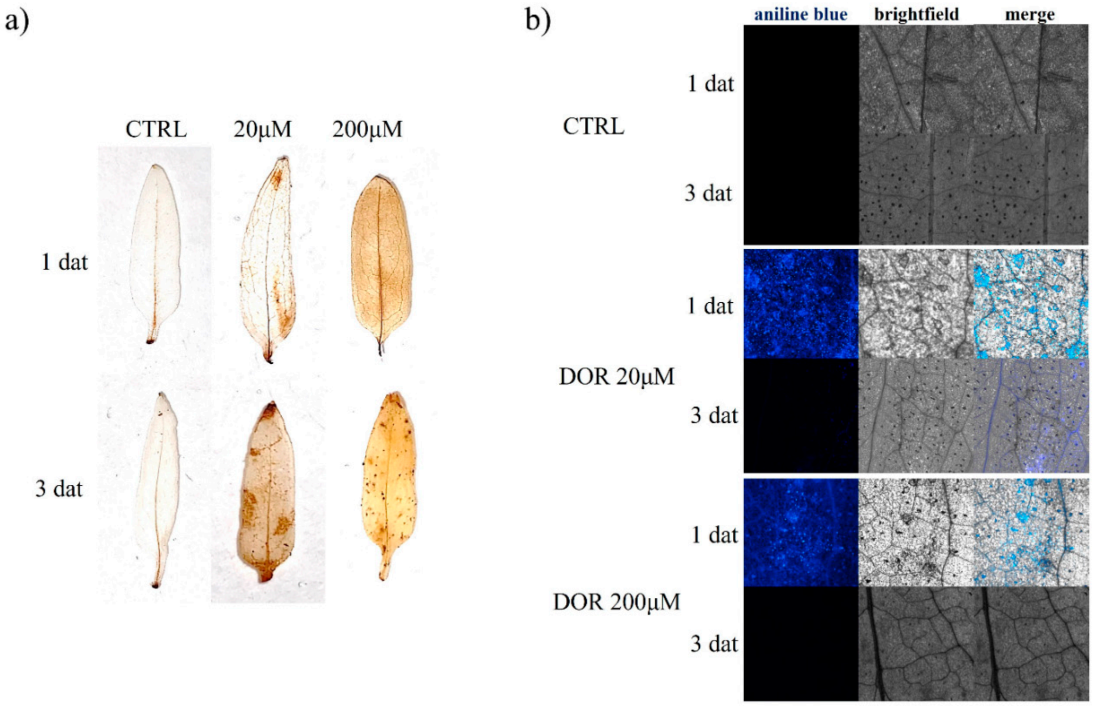

Figure S1. DAB staining (a) and callose deposition (b) of DOR-treated tomato leaves. a) Leaves from two-week-old tomato plants were detached and placed in a 24-well tissue-culture plate with 500 μl of 20 or 200 μM DOR for 1 or 3 days. After treatment, leaves were stained with DAB, as reported in section 3.6 and observed under an optical microscope a t 4x magnification. b) Leaves from two-week-old tomato plants were detached and placed in a 24-well tissue-culture plate with 500 μl of 20 or 200 μM DOR, for 1 or 3 days. After treatment, leaves were stained with 0.01% anilin blue as described in section 3.8 and observed under an optical microscope at 10x magnification. Images are representative of three independent experiments; dat: days after treatment.

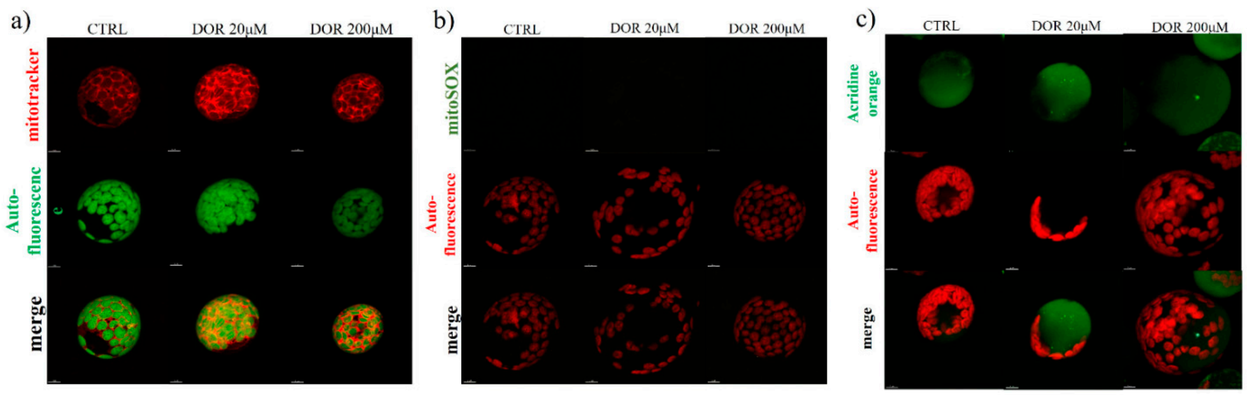

Figure S2. Confocal microscope images of mitochondrial network (a), mitochondria-derived ROS (b) and vacuole (c) of protoplasts treated with DOR. a) Protoplasts from leaves of two-week-old tomato plants were treated with 20 or 200 μM DOR for 1 h, incubated with 1 μM MitoTracker and analyzed under a confocal microscope at 579 nm excitation and 599 nm emission, for mitochondria imaging. b) Protoplasts from leaves of two-week-old tomato plants were treated with 20 or 200 μM DOR for 1 h, incubated with 3 μM MitoSOX and analyzed under a confocal microscope at 510 nm excitation and 580 nm emission, for mitochondrial ROS imaging. c) Protoplasts from leaves of two-week-old tomato plants were treated with 20 or 200 μM DOR for 1 h, incubated with 3 μM acridine orange and analyzed under a confocal microscope at 488 nm excitation and 526 nm emission, for vacuole imaging. Bar = 7 μm.

Table S1. List of oligonucleotides used in qRT-PCR analysis of chloroplast-triggered cell death gene expression of tomato leaves treated with DOR.

Gene	Primer sense 5′-3′	Primer antisense 5′-3′
SolycUBI3	TCTTCCGACACCATCGACAA	AGAACTGCAACACAGTGAGC
SolycACT	CAGCAGATGTGGATCTCAAA	CTGTGGACAATGGAAGGAC
SolycACD2	GCAGTGAACATGACAGTCC	CATGAGAAGGCAGAGTACAAGG
SolycEX1	GAAGAGAAAGCCAGTTCAGC	GCTACGTTGACCTTGAAGCC
